# Imaging the impossible: An fMRI study of impossible causal relationships in magic tricks^☆^

**DOI:** 10.1016/j.neuroimage.2008.12.036

**Published:** 2009-04-15

**Authors:** Ben A. Parris, Gustav Kuhn, Guy A. Mizon, Abdelmalek Benattayallah, Tim L. Hodgson

**Affiliations:** a Exeter Centre for Cognitive Neuroscience, School of Psychology, University of Exeter, Exeter, UK; b Department of Psychology, University of Durham, Durham, UK; c Peninsula MRI research centre, School of Physics, University of Exeter, Exeter, UK

## Abstract

Understanding causal relationships and violations of those relationships is fundamental to learning about the world around us. Over time some of these relationships become so firmly established that they form part of an implicit belief system about what is possible and impossible in the world. Previous studies investigating the neural correlates of violations of learned relationships have focused on relationships that were task-specific and probabilistic. In contrast, the present study uses magic-trick perception as a means of investigating violations of relationships that are long-established, deterministic, and that form part of the aforementioned belief system. Compared to situations in which expected causal relationships are observed, magic trick perception recruited dorso-lateral prefrontal cortex (DLPFC) and anterior cingulate cortex (ACC), brain regions associated with the detection of conflict and the implementation of cognitive control. These activations were greater in the left hemisphere, supporting a role for this hemisphere in the interpretation of complex events. DLPFC is more greatly activated by magic tricks than by surprising events, but not more greatly activated by surprising than non surprising events, suggesting that this region plays a special role in causality processing. The results suggest a role for cognitive control regions in the left hemisphere in a neurobiology of disbelief.

## Introduction

Understanding causal relationships and violations of those relationships is fundamental to learning about the world around us. Whilst for the most part we are unaware of the importance of understanding and perceiving these relationships their importance soon becomes apparent when watching a magic trick in which causal relationships are violated. Imagine the following scenario: A magician places a coin in his right hand and then closes his hand. You are confident the coin is in his right hand; you are as sure as if you had put it there yourself. The magician waves his left hand over his right hand and then slowly opens his right hand to reveal that the coin has disappeared. Your reaction to this magic trick is likely one of astonishment and disbelief. According your implicit system of knowledge the disappearance of the coin should not have been possible, as no force was seen acting upon it. Thus what you have witnessed will violate several causal relationships that you have learnt through past experience. Moreover, you are now likely to try and understand the real causal sequence of events (Kelly, 1980). These violations in causality and expectations are at the heart of magic performances (Kuhn et al., 2008a,b; Kuhn and Land, 2006), and the aim of the present study was to use magic tricks as a tool to investigate the neural correlates of long-established and deterministic causal relationships that form part of a belief system about the world and how it works.

Previous research has used violations in learned associative relations to investigate the cognitive and neural processes underlying causality perception and inference (Fletcher et al., 2001; Fugelsang and Dunbar, 2005; Fugelsang et al., 2005; Hauser and Spaulding, 2006). In one such study (Fugelsang and Dunbar, 2005), the authors investigated the neural roots of biases in causal reasoning. In their study, subjects were asked to interpret data relative to plausible and implausible theories. The plausibility of the theory was manipulated by presenting participants with a brief introductory statement that contained either 1) a direct causal mechanism of action linking a red pill to a mood outcome (e.g. the red pill was described as being able to chemically facilitate the feeling of happiness), or 2) no direct causal mechanism of action linking the red pill to mood outcome (e.g. the red pill was described as being unlikely to chemically facilitate the feeling of happiness). The data were presented such that they were consistent or inconsistent with the causal theory provided; thereby providing instances of both violated and upheld causal relationships. Evaluation of data consistent with a plausible theory recruited neural tissue in the parahippocampal gyrus, which is associated with learning and memory. Evaluating data that was inconsistent with a plausible theory recruited the anterior cingulate cortex (ACC) and left dorso-lateral prefrontal cortex (DLPFC), areas associated with error detection and conflict monitoring (Kerns et al., 2004; Van Veen and Carter, 2006).

In another study involving causality violations, participants learned associations between drugs and syndromes (Fletcher et al., 2001). Activity in the DLPFC was related to learning these associations and also to violations of the learned causal relationships. It was argued that this activity was related to the adaptation of associative relationships in response to unexpected occurrences, and that the activations were surprise-dependent. The present study builds on this research by investigating a different type of causality violation. The associations violated in the studies of Fugelsang and Dunbar (2005) and Fletcher et al. (2001) were task-specific and probabilistic. In the present study, we investigated violations of cause–effect associations that are long-established and deterministic. In doing so, we extend the utility of studying causality violations by relating them to situations in which observers experience disbelief because the violated associations form part of a belief system about possible and impossible causal relationships in the world.

The activation of the DLPFC and the ACC in previous studies of causality violations is important because these brain regions have been implicated in a cognitive control loop in which the ACC is responsible for monitoring for conflict whilst the DLPFC resolves it. The ACC activations in the study by Fugelsang and Dunbar (2005) were interpreted by the authors and subsequently by other researchers (Van Veen and Carter, 2006) as being associated with the detection of conflict between an expected cause and effect relationship and the actual cause and effect relationship that was observed. DLPFC activations were interpreted as the subsequent inferential process; an attempt to resolve the conflict. Similar processes were expected to be activated during magic trick perception. Evidence in support of this would be indicative of a role for these structures in a neurobiology of disbelief.

A causality violation is also a surprising event and so it is likely that the two events share similar neural correlates. However, surprise can follow from events in which there are no violations of causality, suggesting that there are at least some brain regions that are specific to its detection. Studies have implicated ventro-lateral prefrontal cortex (VLPFC; BA45/7) in processing surprising stimuli/events (Braver et al., 2001; Michelon et al., 2003; Parris et al., 2007). These studies have involved the presentation of rare and unusual stimuli, which suggests that VLPFC activations are related to arousal/evaluative mechanisms, whilst DLPFC activations may be related to a deeper level of encoding for the purposes of learning from novel/unexpected events (Fletcher et al., 2001; Fugelsang and Dunbar, 2005; Fugelsang et al., 2005).

In the present study, 25 participants saw 39 video clips while undergoing functional magnetic resonance imaging (fMRI). The clips included 13 magic tricks (the “Magic” condition), as well as 13 clips from each of two control conditions (see Table 1 for a description of selected tricks and controls used in the experiment). An example clip from the Magic condition involves the magician placing a coin in his hand, closing it, and then re-opening it to reveal that the coin is no longer there. The “Causal Control” condition provided a baseline condition that did not involve a causality violation. The Causal Control counterpart to the above example involves the magician putting a coin in his hand and closing it as before, but this time when he opens his hand the coin is still there. The purpose of the “Surprise” condition was to dissociate the detection of causality violations in magic tricks from the detection of alerting or surprising events. In this condition, participants viewed clips of the magician performing an unusual action with one of the objects seen in the magic trick. For example, the magician is seen placing a coin in his hand, but then removing it from his hand and placing it in his mouth.

## Methods

### Participants

25 (17 female) right-handed volunteers ranging in age from 18–34 years (mean age 20.3).

### Stimuli

45 video clips were produced showing either magic tricks (Magic condition), visually similar action sequences without a magic trick (Causal Control condition) or an unusual action with one of the objects seen in the magic tricks (Surprise condition). Each of the 15 clips in each condition were rated by 23 independent observers for how surprising they were, how much they involved illusion, and the extent to which the observed events violated the laws of physical causality, using a scale from 1–7 (7 being the most surprising, involving a strong illusion, and strongly violating causality). Each magic clip was presented in a block with its related clip from the Surprise and Causal Control conditions. The ratings were taken after a single viewing of each clip. On average the 15 video clips used in the Magic condition were rated 4.4 for surprise, 5.4 for illusion, and 4.7 for causality violation. The 15 clips in the Causal Control and Surprise conditions were rated 1.8, 1.3, 1.1 and 2.7, 1.1, 1.1, respectively. Two of the magic tricks were not rated as sufficiently violating cause and effect relationships (rating lower than 2.5) and so these clips and their associated controls were not used in the experiment.

Participants in the fMRI study did not rate the clips. However, the ratings from the independent observers were entered into a reliability analysis to determine the extent to which ratings were likely to be consistent across different groups. All items in all conditions were found to have a Corrected Item-Total Correlation > 0.4, and a Cronbach's Alpha value > 0.9 showing that the item ratings were reliable and consistent across subjects. No item in any condition was found to dramatically improve Cronbach's Alpha value if deleted.

The obtained ratings were used to determine the comparisons made. For example, although the Surprise condition was rated as being less surprising than the Magic condition (*p* < 0.01) it was rated as being significantly more surprising than the Causal Control condition (*p* < 0.001) and thus enabled us to compare the Surprise to the Causal Control condition to identify the neural correlates of surprise. The Magic condition was significantly more surprising, involved greater amounts of illusion and was in greater violation of cause and effect relations than all other conditions (*p* < 0.001).

In order to capture the moment the magic tricks were perceived as such, a further 10 participants who did not take part in the fMRI study were asked to watch the magic tricks and to press a key on a keyboard, as quickly as they could, the moment they realized a magic trick had occurred. Reaction times from these observers were averaged and were used to determine the onset time for event related regressors in the fMRI analysis (see Data analysis).

### Task and procedure

**Fig. 1:**
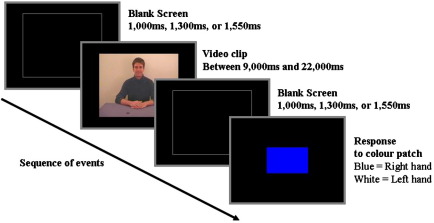
A schematic of the sequence of events in the experiment.

All participants saw all magic clips and their relevant controls once only. 13 clips were used in all conditions. Each magic clip was presented in a block with its related clip from the Surprise and Causal Control conditions. The order of presentation within and between blocks was randomised. Participants were initially presented with the outline of a white rectangle (the same size and shape as the video clips) on a black background. This outline was presented for 1000 ms, 1300 ms, or 1550 ms and was followed by the presentation of a clip. The clips varied in length from 9000–22,000 ms. Following the clip, a blank (black) screen was presented for 1000 ms, 1300 ms, or 1550 ms (see Fig. 1).

To ensure participants remained attentive for the duration of the experiment, participants were given one of two simple tasks to perform following the blank period. Half the participants were presented with a colour patch at fixation for 2000 ms following the blank period and were instructed to press a response key with their right hand if the patch was blue and a different response key with their right hand if the patch was white. The other participants were not presented with the coloured patch but instead saw the question ‘Did you see a magic trick?’ displayed in the centre of the screen for 2000 ms and indicated a Yes/No response by pressing the left/right response key (the mapping of response keys to the Yes/No decision was randomized across subjects). These two task types were included because we were interested in whether the experimental setting altered the experience of magic trick perception. By setting two tasks, one which of was designed to encourage participants to adopt an attentional set in which they actively looked out for magic tricks on each trial (responding to the question), we were able to assess whether attentional set influences the activations observed in the experiment. Between-subjects *t*-tests revealed that although some activation differences were observed between the two judgment tasks (in the lentiform nucleus (24 − 7 18) and the cuneus (− 12 − 71 16) for the Magic condition and the middle occipital gyrus (− 30 − 84 − 1) for the Causal Control condition), no differences were observed in a priori regions of interest (deemed to be of interest based on previous neuroimaging studies; see fMRI analysis section below) such as the DLPFC which has been associated with reasoning. The finding of no differences in a priori areas between the two tasks suggests that magic trick perception recruits similar brain regions under differing attentional sets. For this reason, the data reported below are collapsed across task type.

### fMRI data acquisition

Scanning was performed on a 1.5 T Philips Gyroscan magnet at the Peninsula MRI research centre, University of Exeter, UK. A T2⁎-weighted echoplanar sequence was used (TR = 3000 ms, TE = 50 ms, flip angle 90°, 32 transverse slices, 3.6 × 3.6 × 4 mm, ascending acquisition). 350 volumes were acquired per subject. An additional 5 “dummy” scans were performed at the start of each block prior to the start of the stimulus sequence.

### Data analysis

Data were analysed using SPM2 software (www.fil.ion.ucl.ac.uk/spm). The images were realigned, unwarped to remove variance caused by movement-by-field-inhomogeneity interactions, normalised to a standard EPI template, and smoothed with a Gaussian kernel of 6 mm full-width at half maximum. Statistical regressors were generated by convolving a canonical hemodynamic response function with a series of discrete event onset times (0 ms duration), time locked to the presentation of video clips in the Magic, Causal Control and Surprise conditions. The exact timing of each event onset relative to clip onset times was based on the reaction time of magic/non magic judgments obtained from viewers prior to scanning (see Stimuli above). A general linear model approach was used to estimate parameter values for each regressor. The analyses generated a series of “t contrast images” for each effect and subject which were entered into a 2nd level (“random effects”) analysis consisting of one-sample t-tests with a hypothesised mean of 0 (thresholded at *p* = 0.001, uncorrected). To further protect against the probability of type 1 error, we employed an extent voxel threshold cut-off of 30. This combination of intensity and extent thresholds produces a per voxel false positive probability of < 0.000001 (Forman et al., 1995). Two sample repeated measures t-tests with a statistical threshold of *p* < 0.001, uncorrected, and a voxel cluster size threshold of 30 were also performed on the Magic > Causal Control, Magic > Surprise, and Surprise > Causal Control. These analyses were confined to regions of interest (ROIs) as defined by the WFU Pickatlas tool (Maldjian et al., 2003). The ROIs chosen were the frontal, parietal, and limbic lobes and were selected on the basis of having been implicated previously in studies investigating detecting causality violations (Fletcher et al., 2001; Fugelsang and Dunbar, 2005). The *X*,*Y*,*Z* coordinates of all activation clusters were transformed from normalized MNI space (i.e. SPM coordinates) to Talairach space (www.mrc-cbu.cam.ac.uk/Imaging/mnispace.html) in order to ascertain the site of activation relative to the atlas of Talairach and Tournoux, (1988).

## Results

**Fig. 2:**
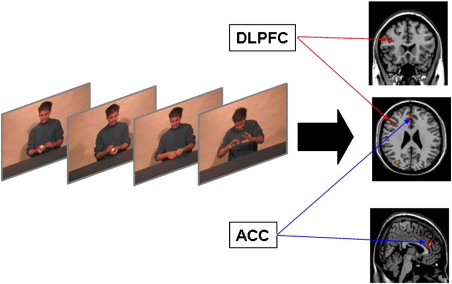
A comparison between Magic and Causal Control conditions revealed activations in left dorsolateral prefrontal cortex (DLPFC; *x*, *y*, *z* Talairach coordinates − 42 23 23) and left dorsal anterior cingulate cortex (ACC; − 4 38 16), cognitive control regions in the left hemisphere suggesting a role for these regions in a neurobiology of disbelief.

To identify neural activations associated with violations of causality a paired-samples *t*-test between the Magic and Causal Control conditions was performed (see Table 2 for a list of activation sites from all comparisons). This contrast yielded significantly greater activations in three areas in left DLPFC including the superior frontal gyrus (BA6), and two areas on the middle frontal gyrus (BA8, BA46; see Fig. 2). Furthermore, significantly greater activations were observed in the left dorsal ACC (BA32). These areas are consistent with previous studies investigating the neural correlates of causality violations (Fletcher et al., 2001; Fugelsang and Dunbar, 2005). However, since the present study involves violations of long-established, deterministic associations, these activations suggest that ACC and DLPFC in the left hemisphere play a role in the neurobiology of disbelief.

**Fig. 3:**
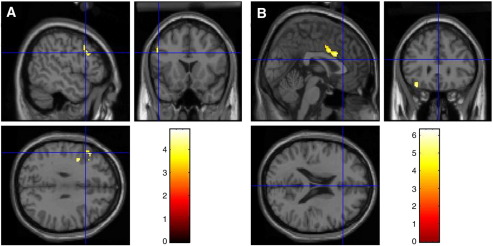
(A) A comparison between the Magic and Surprise conditions revealed activations in left dorso-lateral prefrontal cortex (*x*, *y*, *z* Talairach coordinates, − 42 24 23). (B) A comparison between the Surprise and Causal Control condition reveals activations in ventral anterior cingulate cortex (*x*, *y*, *z* Talairach coordinates, 2 19 25) and left ventral prefrontal cortex (− 31 30 − 15), but not in DLPFC, further supporting the inference that DLPFC plays special role in detecting violations of causality.

To determine which neural regions were recruited by surprising events a comparison was made between the Surprise and Causal Control conditions (see Fig. 3A). The analysis revealed significantly greater activations in left VLPFC (inferior frontal gyrus; BA47), left dorso-medial PFC (superior frontal gyrus; BA9), right ACC (BA24), and the left precuneus (BA7). These results support a role for ventral and dorsal regions of the prefrontal cortex in the processing of surprising stimuli/events (Braver et al., 2001; Parris et al., 2007). As with the comparison between the Magic and the Causal Control conditions, ACC and PFC showed activations in the present comparison, suggesting that the same regions are activated in situations in which expectancies, and not just causal relations are violated. Importantly, however, no activations were observed in DLPFC for this comparison, suggesting this region plays a special role in processing causality violations.

To identify which of the neural regions implicated in magic trick perception plays a special role in processing causality violations, a comparison was made between the Magic and Surprise conditions (see Fig. 3B). The comparison revealed significantly greater activations in left DLPFC (BA46), right dorso-medial PFC (BA9), right DLPFC (BA10), right precuneus (BA19), and further areas in the parietal lobe bilaterally (BA40). Of particular relevance is left DLPFC; this area has not been implicated in processing surprising events in this study, but has been implicated in magic trick perception. This again suggests that left DLPFC (BA46) plays a special role in processing violations in causality.

## Discussion

In contrast to previous neuroimaging studies of violations of causality the present study violated causal associations that were deterministic and long-established, rather than probabilistic and task-specific. In comparison to events in which causal relations were upheld, magic trick perception was associated with greater activation in left dorso-lateral prefrontal and left anterior cingulate cortices. In Fugelsang and Dunbar's (2005) study involving causality violations, ACC activations were interpreted as being associated with the detection of conflict between the expected and observed causal relations whilst the DLPFC activations were interpreted as being associated with reasoning about the observed events, since their activations were greater in the left hemisphere, which has been shown to be involved in interpreting complex actions and events (Gazzaniga, 2000; Roser et al., 2005). Attributing such roles to the ACC and left DLPFC is also possible in the present study, and indeed the violation of causality observed in magic tricks can be considered similar to Fugelsang and Dunbar's theory-inconsistent condition in that participants will have had a prior theory or belief about what causal relationships are possible or not. Left hemisphere activity in this context may represent an attempt to assign a cause to the effects observed in magic tricks and as such may be seen to represent an attempt to resolve the conflict between what is observed and what is thought to be possible.

Previous research has shown that ACC and DLPFC are activated in situations of response conflict, in which the relevant and irrelevant dimensions of a stimulus activate conflicting responses (Kerns et al., 2004; MacDonald et al., 2000). Under these conditions the ACC is thought to detect the conflict and the PFC implicated in resolving it (Kerns et al., 2004; Van Veen and Carter, 2006). An important question remains, however, as to whether this control loop plays a wider role in cognitive phenomena (Van Veen and Carter, 2006). The present results indicate a role for these regions in detecting conflict resulting from the comparison of beliefs with observed events (note that the activations reported in our study could not index response conflict), supporting the possibility that these structures play a wider role in the architecture of cognition. In line with this finding, a recent study separated the decision and response phases of a decision task, and showed that ACC also indexes conflict at the decision stage (Ponchon et al., 2008).

Despite observing DLPFC activity, Fletcher et al. (2001) did not observe activity in ACC in their study when expected causal sequences were violated. However, Fugelsang and Dunbar (2005) did, but only when the violated associative causal relation was underpinned by a plausible theory linking the cause and the effect. Presumably the plausible theory increased the magnitude of the violation by embedding the causal relation within a knowledge base. Our results also show ACC activation in response to a violation of strong causal relations, raising the possibility that only those causal relationships bound by a theory or belief appear to activate ACC when violated.

Importantly, the ACC and the PFC were also activated by surprising events. Both ventro-lateral PFC (VLPFC) and ACC activations were observed in the comparison between the Surprise and the Causal Control conditions. Since both magic tricks and surprising events involve violations of expectancies, these results suggest that the cognitive control loop may be activated when there is strong conflict between expectations and observed events. Such a role is consistent with the strong connections between ACC and sensori-motor cortices because unexpected events often require the flexible control of action. Although ACC has not previously been implicated in processing surprise, the veridical, real-world nature of the surprising events presented in the present study (i.e. real physical manipulation of 3D objects, rather than abstract computer-generated stimuli) might have resulted in stronger surprise responses than those elicited in other studies involving unexpected events/stimuli (Braver et al., 2001; Michelon et al., 2003; Parris et al., 2007). Critchley (2005) and Critchley et al. (2003, 2005) have observed that ACC activity closely predicts measures of autonomic arousal and have suggested that ACC regulates autonomic arousal to accommodate cognitive tasks and thus would be expected to be observed in response to alerting events. This theory of ACC function is not incompatible with the notion that it is important in the detection of conflict (Carter and Van Veen, 2007). In addition, this theory accounts well for the possibility that ACC activity appears to be related to the strength of the violated causal relationship in that the stronger the causal relationship the more alerting or arousing would be a violation of that relationship.

The capacity to detect information that contradicts or challenges an established system of knowledge is crucial for learning about the world around us. The notion that ACC activity is a function of the extent of the contradiction suggests it that it might play a role in a neurobiology of disbelief. A recent study has shown ACC and PFC activation when defining a propositional statement as false (e.g. ‘Wisconsin is on the west coast of the United States’; Harris et al., 2008). These activations were not observed when defining propositional statements as true. These results suggest that these structures play a role in establishing the veracity of information in the sense of registering whether the information conflicts with pre-existing knowledge. Magic trick perception likewise involves a strong notion that what is observed is impossible and must therefore be false and disbelieved. Taken together, these results suggest that PFC and ACC activity might play an important role in learning from situations that conflict with existing knowledge, situations one would expect would lead to the experience of disbelief or a judgment of falsity. However, an equally relevant finding from Harris et al. is that ACC activation was also prominent when participants were uncertain about the veracity of a statement, suggesting that the ACC activity observed in the present study could be due to the likely experience of being uncertain about what was observed after perceiving a magic trick.

The finding of ACC and PFC activity in both the Magic v Causal Control comparison and the Surprise v Causal Control comparison is mitigated by the finding that different parts of the PFC were associated to these two event types: Dorso-lateral PFC with magic trick perception, and, consistent with previous research, ventro-lateral PFC with the perception of surprise events that do not involve a causality violation (Braver et al., 2001; Parris et al., 2007). This result indicates that VLPFC activations are related to arousal/evaluative mechanisms, whilst DLPFC activations may be related to a deeper level of encoding for the purposes of learning from novel/unexpected events. It is also important to note that whilst violations of causality can be seen as a subset of violations of expectancy — in that a causality violation generally violates our expectancies — our results suggest that brain areas responsible for detecting expectancy violations in general (i.e. ACC and VLPFC) are not responsible for detecting causality violations in particular; this function is specific to DLPFC. Interestingly, the right inferior middle frontal gyrus locus of activity specific to the Magic versus Surprise comparison lays within BA10, an area thought to play a role in switching attention between stimulus-oriented and stimulus-independent thought (e.g. Gilbert et al., 2005). Detection of causality violations may require both attention to an external stimulus and its evaluation relative to an internal representation of beliefs about the world.

The inclusion of the Surprise condition in the present study represents an improvement over previous studies investigating causality violations because it allows us to dissociate the detection of causality violations from the detection of alerting or surprising events. However, the Magic condition was rated as being significantly more surprising than our Surprise condition which means that left DLPFC activity observed in the Magic v Surprise comparison could be due to greater levels of surprise in the Magic condition. This possibility is rendered less likely given that left DLPFC was not more greatly activated by the Surprise than the Causal Control condition. In addition, our results indicate a special role for VLPFC in processing alerting or surprising events. Nevertheless, future studies need to further investigate whether the neural regions underlying causality violations dissociate from those involved in the detection of surprising events before any strong conclusions can be drawn.

One final implication of the present results relates to hemispheric specialization. Gazzaniga (2000) proposed that the left hemisphere is responsible for interpreting complex stimuli and actions. Exploring this idea Roser et al. (2005) tested two callosotomy patients on tasks involving causal perception and causal inference. Their results showed that the direct perception of causality and the ability to infer causality depend on different cerebral hemispheres; the right and left hemispheres, respectively. In the present study the Magic vs. Causal Control comparison produced activations in the left hemisphere only suggesting that these activations in some way reflect an inference process. The present experiment did not require participants to report on their thoughts after seeing the magic trick so we cannot make any strong conclusions as to the nature of the processes reflected in these activations, but such clear hemispheric lateralization, which was also observed in a post-hoc whole brain analysis (in fact there were no additional areas of activation in the whole brain analysis of this comparison), is unusual and suggestive of a unique function associated with left hemisphere processing such as interpreting complex stimuli and events (Gazzaniga, 2000) and reasoning (e.g. Goel et al., 1998).

The results from the present study show that DLPFC and ACC are activated by conflict between expected and observed events. The notion that they are involved in detecting conflict in this broad sense is important for understanding the importance of these structures in our cognitive architecture (Van Veen and Carter, 2006). These activations were confined to the left hemisphere, supporting a role for this hemisphere in the interpretation of complex events. DLPFC is more greatly activated by magic tricks than by surprising events, but not more greatly activated by surprising than unsurprising events, suggesting that this region plays a special role in causality processing. The impossible nature of the causality violations used in the present experiment suggests a role for cognitive control regions in the left hemisphere in a neurobiology of disbelief.

## Figures and Tables

**Table 1:** Examples of the clips seen in the experimental conditions

	Magic condition	Causal control condition	Surprise condition
	*A cause–effect relationship is violated*	*The cause–effect relationship violated in the magic trick is upheld*	*Something unusual and unexpected happens, but cause–effect relations are upheld*
Disappearing coin	A coin is placed on the table and is covered by the magician's hand. The magician appears to rub the coin into the table. He lifts his hand to reveal that it has disappeared.	A coin is placed on the table and is covered by the magician's hand. The magician appears to rub the coin into the table. He lifts is hand to reveal the coin.	A coin is placed on the table and is covered by the magician's hand. The magician appears to rub the coin into the table. He lifts his hand to reveal the coin. A third hand then swipes in and removes the coin.
Disappearing glass	A glass is covered with a napkin. The magician hits the top of the napkin which immediately crumples to the table. The glass has disappeared.	A glass is covered with a napkin. The magician hits the top of the napkin which maintains the shape of the glass underneath.	A glass is covered with a napkin. The magician pushes the napkin covered glass off the table.
Disappearing silk	The magician pushes a silk handkerchief into his closed hand. The closed hand opens to reveal the handkerchief has disappeared.	The magician pushes a silk handkerchief into his closed hand. The closed hand opens to reveal the handkerchief.	After a similar beginning, the magician unexpectedly puts a silk handkerchief in his mouth. He completes the sequence with the ‘end of trick’ hand gesture (showing his palms to the observer).
Levitation	The magician crumples up a paper napkin and places it in his hand. He then moves his hand downwards whilst the napkin remains in its place, apparently levitating.	The magician crumples up a paper napkin and places it in his hand. He then moves his hand downwards; the napkin moves with the hand.	The magician crumples up a paper napkin and places it in his hand. He then unexpectedly flicks the napkin from his hand. He completes the sequence with the ‘end of trick’ hand gesture.
Changing bill	A £5 note is folded and unfolded. Upon unfolding it is revealed that the £5 note is now a £10 note.	A £5 note is folded and unfolded.	A 5 pound note is torn in two. He completes the sequence with the ‘end of trick’ hand gesture.
Torn and restored cigarette	The magician tears a cigarette into two pieces and pushes the pieces through his hand and emerges restored	The magician tears a cigarette into two pieces and pushes the pieces through his hand and emerges in pieces.	The magician uses the cigarette like a comb.

**Table 2:** Comparisons: paired-sample *t*-tests, voxel cluster threshold 30, *p* < 0.0001, uncorrected

	*X*	*Y*	*Z*	*T*-value	*Z*-value	Neuroanatomical location
Magic > Causal Control	− 24	12	53	5.66	4.47	Left superior frontal gyrus BA6
− 22	37	39	4.50	4.21	Left middle frontal gyrus BA8
− 42	23	23	4.69	3.91	Left middle frontal gyrus BA46
− 4	38	16	5.53	4.40	Left anterior cingulated BA32
Magic > Surprise	− 42	24	23	4.86	4.02	Left middle frontal gyrus BA46
4	33	32	4.76	3.95	Right medial frontal gyrus BA9
34	52	− 1	4.61	3.86	Right middle frontal gyrus BA10
− 44	− 46	52	6.20	4.74	Left inferior parietal lobe BA40
44	− 46	54	5.90	4.59	Right inferior parietal lobe BA40
26	− 72	35	5.38	4.32	Right precuneus BA19
− 32	− 49	32	5.08	4.14	Left supramarginal gyrus BA40
− 31	30	− 15	6.32	4.80	Left inferior frontal gyrus BA47
Surprise > Causal Control	− 4	56	30	4.77	3.96	Left superior frontal gyrus BA9
− 4	− 50	41	4.55	3.83	Left precuneus BA7
2	19	25	5.64	4.46	Right anterior cingulated BA24
